# Manure and Doxycycline Affect the Bacterial Community and Its Resistome in Lettuce Rhizosphere and Bulk Soil

**DOI:** 10.3389/fmicb.2019.00725

**Published:** 2019-04-16

**Authors:** Khald Blau, Samuel Jacquiod, Søren J. Sørensen, Jian-Qiang Su, Yong-Guan Zhu, Kornelia Smalla, Sven Jechalke

**Affiliations:** ^1^Julius Kühn-Institut—Federal Research Centre for Cultivated Plants, Institute for Epidemiology and Pathogen Diagnostics, Braunschweig, Germany; ^2^Section of Microbiology, Department of Biology, University of Copenhagen, Copenhagen, Denmark; ^3^Key Lab of Urban Environment and Health, Institute of Urban Environment, Chinese Academy of Sciences, Xiamen, China; ^4^State Key Lab of Urban and Regional Ecology, Research Center for Eco-Environmental Sciences, Chinese Academy of Sciences, Beijing, China

**Keywords:** manure, bulk soil, lettuce rhizosphere, resistance genes, prokaryotic community, high-throughput quantitative polymerase chain reaction

## Abstract

Manure application to agricultural soil introduces antibiotic residues and increases the abundance of antibiotic-resistant bacteria (ARB) carrying antibiotic resistance genes (ARGs), often located on mobile genetic elements (MGEs). The rhizosphere is regarded as a hotspot of microbial activity and gene transfer, which can alter and prolong the effects of organic fertilizers containing antibiotics. However, not much is known about the influence of plants on the effects of doxycycline applied to soil via manure. In this study, the effects of manure spiked with or without doxycycline on the prokaryotic community composition as well as on the relative abundance of ARGs and MGEs in lettuce rhizosphere and bulk soil were investigated by means of a polyphasic cultivation-independent approach. Samples were taken 42 days after manure application, and total community DNA was extracted. Besides a pronounced manure effect, doxycycline spiking caused an additional enrichment of ARGs and MGEs. High-throughput quantitative PCR revealed an increase in tetracycline, aminoglycoside, and macrolide–lincosamide–streptogramin B (MLSB) resistance genes associated with the application of manure spiked with doxycycline. This effect was unexpectedly lower in the rhizosphere than in bulk soil, suggesting a faster dissipation of the antibiotic and a more resilient prokaryotic community in the rhizosphere. Interestingly, the tetracycline resistance gene *tetA*(P) was highly enriched in manure-treated bulk soil and rhizosphere, with highest values observed in doxycycline-treated bulk soil, concurring with an enrichment of Clostridia. Thus, the gene *tetA*(P) might be a suitable marker of soil contamination by ARB, ARGs, and antibiotics of manure origin. These findings illustrate that the effects of manure and doxycycline on ARGs and MGEs differ between rhizosphere and bulk soil, which needs to be considered when assessing risks for human health connected to the spread of ARGs in the environment.

## Introduction

Antimicrobial resistance is a serious public health problem that threatens the achievements of modern medicine (WHO, 2014). Enormous amounts of veterinary antibiotics have been used worldwide in animal husbandry to promote growth and to treat or prevent bacterial diseases (Arikan et al., 2007). Therefore, manure application to agricultural soils introduces not only a mixture of different nutrients and organic matter that improves the growth of plants (Aust et al., 2009) but also antibiotic-resistant bacteria (ARB) carrying antibiotic resistance genes (ARGs), often located on mobile genetic elements (MGEs), together with residuals of veterinary antibiotic compounds and their degradation products (Sarmah et al., 2006; Heuer and Smalla, 2007; Ding et al., 2014; Jechalke et al., 2014; Wolters et al., 2016, 2018; Yang et al., 2017). A number of studies have demonstrated that manure-derived ARB and their ARGs may persist in soil for a few weeks and up to years and that these ARGs can be horizontally transferred into native soil bacteria (Haack and Andrews, 2000; Chee-Sanford et al., 2001; Schmitt et al., 2006; Heuer and Smalla, 2007; Heuer et al., 2008, 2011a; Binh et al., 2009; Jechalke et al., 2013b; Udikovic-Kolic et al., 2014; Wichmann et al., 2014). Furthermore, the application of antibiotic-containing manure to agricultural soils can result in increasing levels of ARGs and the selection of resistant pathogens (Heuer and Smalla, 2007; Schwaiger et al., 2009; Heuer et al., 2011a; Looft et al., 2012; Wellington et al., 2013). This antibiotic selection pressure is assumed to play a key role in the spread of ARGs by means of vertical and horizontal gene transfer (HGT). Thus, several studies have shown that sub-therapeutic levels of antibiotics, as commonly observed in soil, promote the abundance of ARB, because these concentrations of antibiotics can provide a selective advantage to ARB, thus leading to shifts in the composition of bacterial communities (Ghosh and LaPara, 2007; Gullberg et al., 2011; Alexander et al., 2013; Xiong et al., 2015) and even to the development of high level resistance (Wistrand-Yuen et al., 2018).

Tetracyclines, discovered in the 1940s, are among the most commonly used antibiotics in animal farming (Chopra and Roberts, 2001) and have long been used against a wide range of Gram-negative and Gram-positive bacterial pathogens (Chopra and Roberts, 2001; Chee-Sanford et al., 2009). Doxycycline, a member of the tetracycline class of antibiotics, was frequently detected in manure (Tylová et al., 2010; Carballo et al., 2016; van den Meersche et al., 2016; Widyasari-Mehta et al., 2016; Wolters et al., 2016; Albero et al., 2018). In a previous study, we could demonstrate that the application of high doses of doxycycline-spiked manure to agricultural soils was correlated with an enrichment of ARGs and class 1 integrons and with changes in the soil bacterial community composition, more pronounced in sandy compared to loamy soil (Blau et al., 2017). However, only a small selection of genes was quantified and changes in the bacterial community were assessed by denaturing gradient gel electrophoresis (DGGE), not providing qualitative information on potential responders to the antibiotic spiked to manure. Furthermore, it is not known so far if plants can alter the fate and effects of doxycycline in the rhizosphere and phyllosphere compared to bulk soil, which should be considered when assessing associated risks for human health.

In the rhizosphere of plants, physicochemical properties and the availability of root exudates are assumed to be important drivers for the development of a plant-specific microbiota (Smalla et al., 2001; Schreiter et al., 2014). Furthermore, the plant rhizosphere is considered to be a hotspot of HGT, which seems to be modulated by root growth and exudates production, increasing microbial abundance and activity (van Elsas et al., 2003; Mølbak et al., 2007; Heuer and Smalla, 2012). Accordingly, it was observed in previous studies that the fate and effects of antibiotics in the rhizosphere can differ from bulk soil (Rosendahl et al., 2011; Jechalke et al., 2013a,b; Chen et al., 2018). However, this was not shown so far for doxycycline. More importantly, ARB and residual antibiotics derived from manure amendments to soil could foster the transfer of ARGs into bacterial communities associated to crops, ultimately posing the risk of antibiotic resistance transmission to the human microbiota.

In the present study, a greenhouse experiment was performed to determine the effects of doxycycline-spiked manure on the resistome, MGEs, and the soil prokaryotic community composition in bulk soil and lettuce rhizosphere. Therefore, selected ARGs and MGEs were quantified in rhizosphere, bulk soil, and manure, as well as in the phyllosphere by the use of conventional quantitative real-time PCR (TaqMan-based). Additionally, the abundance and diversity of ARGs and MGEs were analyzed by high-throughput quantitative PCR (HT-qPCR) with 296 primer sets including 285 ARGs, 8 transposase genes, 2 class 1 integron integrase genes, and the bacterial 16S rRNA gene, and the results of the two approaches were compared regarding their sensitivity. In parallel, effects on the prokaryotic community composition in bulk soil and rhizosphere were analyzed by Illumina MiSeq sequencing of 16S rRNA genes, amplified from TC-DNA.

## Materials and Methods

### Properties of Soil and Manure

The soil used for the experiment was silty sand obtained from Landwirtschaftliche Untersuchungs- und Forschungsanstalt Speyer (LUFA Speyer, Speyer, Germany ^1^). The soil has not been treated with manure, pesticides, or biocidal fertilizers for at least five years. The collected soil was air-dried and sieved with a pore diameter of 2 mm. The physicochemical properties of the soil used in this study were as follows: pH value (0.01 M CaCl_2_) 4.9, organic carbon 0.71%, nitrogen 0.06%, and cation-exchange capacity 4.2 (meq/100 g). Manure was obtained from a pig husbandry farm in Lower Saxony, Germany. The collected manure was stored at 4°C until the beginning of the experiment. The concentration of doxycycline in the manure was below 1 mg kg^-1^ wet weight (Blau et al., 2017). The manure was characterized by the LUFA Nord-West (Hameln, Germany). It had a dry weight of 3.8%, total N 10.1%, NH_4_^+^ 4.7%, P 3.6%, K 8.1%, Mg^2+^ 1.0%, Ca 6.2%, and S 1.3%. Furthermore, the manure contained 664.1 mg Cu kg^-1^ and 1,998 mg Zn kg^-1^. Manure samples were collected in triplicate, placed in 15-ml falcon tubes, and stored at -20°C until DNA extraction was performed.

### Experimental Design

The experiment was conducted in a greenhouse. The microcosms were set up using polypropylene pots, each containing 300 g of soil (dry weight). Each treatment included three replicates and was designated as (i) untreated bulk soil (BS-C) and rhizosphere (R-C), (ii) manure-treated bulk soil (BS-M) and rhizosphere (R-M) without antibiotic, and (iii) manure-treated bulk soil and rhizosphere supplemented with doxycycline (50 or 100 mg kg^-1^ soil dry weight) (BS-M-D50 or BS-M-D100 and R-M-D50 or R-M-D100, respectively). Doxycycline stock solution (Doxycycline Hyclate, purity > 98%, Sigma-Aldrich, Munich, Germany) was prepared by dissolving the compound in water with subsequent sterile filtering (0.2 μm). Forty grams of manure per kilogram of soil was applied as previously described, corresponding to a realistic input according to agricultural practice (Heuer et al., 2011b). Before mixing with the soil, the manure was spiked with the antibiotic stock solution. Untreated and manure-treated soils served as controls to assess the effects of manure and of doxycycline, respectively.

The lettuce (*Lactuca sativa* L. cv. Tizian) seeds were sown in small pots and pre-grown for four weeks. The seedlings in the three-leaf stage were transplanted to the pots with the different soil treatments, directly after mixing the soil with manure or doxycycline-spiked manure. All microcosms were incubated for 6 weeks at 20°C in the greenhouse with 16-h light. Water was added as needed, and the pots were sampled on day 42.

### Sampling of Bulk Soil, Lettuce Rhizosphere, and Phyllosphere

On day 42, lettuce rhizosphere and bulk soil samples were taken by removing the lettuce plants from the soil. Loosely bound soil was shaken off the roots. The soil sticking to the roots after shaking was considered rhizosphere. The residual soil in the pots was thoroughly mixed and was considered bulk soil. One gram of bulk soil or root with adhering rhizosphere soil was weighed and transferred into centrifugation tubes (50 ml). The prokaryotic cells were extracted by adding 3 ml of sterile 0.3% NaCl solution and mixing on the vortex. This step was repeated three times, and the supernatants (3 × 3 ml) were combined in new 50-ml centrifugation tubes and centrifuged at 3,100 ×*g* for 15 min at 4°C. The pellets were stored at -20°C until DNA extraction.

Phyllosphere samples were obtained by cutting the leaves with a sterile scalpel and weighing 5 g of the cut samples in to sterile Stomacher^®^ bags. The samples were mixed with 15 ml of 0.3% NaCl solution three times and homogenized for 1 min at high speed using a Stomacher^®^ 400 (Seward, Worthing, United Kingdom). The supernatants were combined and centrifuged at 3,100 ×*g* for 15 min at 4°C. The pellets were stored at -20°C until DNA extraction.

### DNA Extraction and Purification

Total community (TC)-DNA was extracted from 0.1 g of homogenized soil or rhizosphere pellets and from 0.1 g of manure by using the FastDNA^®^SPIN Kit for soil (MP Biomedicals, Heidelberg, Germany) according to the manufacturer’s instructions. The phyllosphere pellets were taken as a whole and DNA extraction was performed as described for the bulk soil and rhizosphere pellets. The extracted TC-DNA samples were purified using the GeneClean^®^ Spin Kit (MP Biomedicals), following the manufacturer’s recommendations. The quality of extracted DNA was determined by agarose gel electrophoresis. The extracted DNA was stored at -20°C until further analysis.

### Detection and Quantification of Target Genes via Real-Time qPCR

Selected genes were quantified in TC-DNA from manure, bulk soil, rhizosphere, and phyllosphere by quantitative real-time PCR 5′-nuclease assays (TaqMan qPCR) in a CFX96 real-time PCR detection system (Bio-Rad, Hercules, CA, United States). The targeted genes included *intI1* and *intI2* for class 1 and 2 integron integrase genes; *korB* specific for IncP-1 plasmids; *qacE* and/or *qacEΔ1* (*qacE*/*qacEΔ1*) encoding quaternary ammonium compound resistance; *aadA* encoding streptomycin and spectinomycin resistance; *tet*(W), *tet*(Q), *tet*(A), and *tet*(M) encoding tetracycline resistance; and *sul1* encoding sulfonamide resistance. The 16S rRNA genes were quantified by using the primers BACT1369F and PROK1492R and the probe TM1389F. The primers, qPCR conditions, and references are listed in Supplementary Table S1. The relative abundances of ARGs and MGEs were calculated as copy number of ARG or MGE divided by the copy number of 16S rRNA genes and subsequent log transformation. Due to co-extracted plant DNA interfering with the quantification of the bacterial 16S rRNA genes, for the phyllosphere samples, the absolute abundances of the ARGs were determined and expressed as gene copies per gram of leaf. The Pearson correlation coefficient (r) and *p*-values between the relative abundance of ARGs and concentrations of antibiotics were tested by using the CORR procedure of the SAS statistical package (*p* < 0.05; SAS 9.3; SAS Institute, Inc., Cary, NC, United States). Comparisons between pairs of data were made using the GLIMMIX procedure (Tukey test, *p* < 0.05; SAS 9.3).

### High-Throughput Quantitative PCR

HT-qPCR was performed for the purified TC-DNA extracted from bulk soil and rhizosphere treatments (BS-C, BS-M, BS-M-D100, and R-M-D100) as well as from one manure sample. Therefore, the HT-qPCR was performed using the WaferGen SmartChip Real-time PCR system (WaferGen Bio-systems, Inc., United States) as described previously (Su et al., 2015) with slight modifications. Each PCR reaction (100 nl each well) consisted of 1 × LightCycler 480 SYBR Green I Master (Roche Applied Sciences, Indianapolis, IN, United States), 1 mg ml^-1^ bovine serum albumin (New England Biolaboratories, Beverly, MA, United States), 500 nM of each primer, and DNA template of 2.5 ng μl^-1^. In total, 296 primer sets were used targeting 284 ARGs for major classes of antibiotics, 8 transposase genes, 2 class 1 integron integrase genes (including the clinical class 1 integron), the *bla*_NDM-1_ gene, and the 16S rRNA gene (Su et al., 2015).

The results of the qPCR runs were pre-processed as described previously, amplifications beyond the range of 90–110% were discarded, and only amplifications in all three technical replicates were considered positive (Su et al., 2015). The gene copy number was estimated (Eq. 1) as described recently (Ouyang et al., 2015). The fold change compared to the controls was calculated by a comparative CT method (Eqs 2–4) as previously described (Schmittgen and Livak, 2008; Zhu et al., 2013). For the calculation of fold changes, controls below the detection limit of threshold cycle (CT) 31 or without amplification were replaced by CT 31. For soil, the detection of enriched genes in one treatment only or shared within treatments was visualized by a Venn diagram created by using the R package “VennDiagram.”

$$\begin{array}{l} \text{Gene copy number}\;=\;10^{\left(({\text{31-C}}_{\text{T}})\text{/}(\text{10/3})\right)}\;\;\;\;\;\;\;\;\;\;\;\;\;\;\;\;\;\;\;\;\;\;\;\;\;\;\;\;\;\left(1\right) \end{array}$$

$$\begin{array}{l} \Delta {\text{C}}_{\text{T}}\;\text{=}\;{\text{C}}_{\text{T}(\text{ARG})}{\text{-C}}_{\text{T}(\text{16S})}\;\;\;\;\;\;\;\;\;\;\;\;\;\;\;\;\;\;\;\;\;\;\;\;\;\;\;\;\;\;\;\;\;\;\;\;\;\;\;\;\;\;\;\;\;\;\;\;\;\;\;\;\;\;\;\;\;\;\;\;\;\;\;\;\;\;\;\;\;\;\;\;\;\;\;\left(2\right) \end{array}$$

$$\begin{array}{l} \Delta \Delta {\text{C}}_{\text{T}}\;\text{=}\;\Delta {\text{C}}_{\text{T}(\text{Treatment})}\text{-}\Delta {\text{C}}_{\text{T}(\text{Control})}\;\;\;\;\;\;\;\;\;\;\;\;\;\;\;\;\;\;\;\;\;\;\;\;\;\;\;\;\;\;\;\;\;\;\;\;\;\;\;\;\;\;\;\;\;\;\left(3\right) \end{array}$$

$$\begin{array}{l} \text{Fold change}\;=\;2^{\left(-\Delta \Delta {\text{C}}_{\text{T}}\right)}\;\;\;\;\;\;\;\;\;\;\;\;\;\;\;\;\;\;\;\;\;\;\;\;\;\;\;\;\;\;\;\;\;\;\;\;\;\;\;\;\;\;\;\;\;\;\;\;\;\;\;\;\;\;\;\;\;\;\;\;\;\;\;\;\;\;\left(4\right) \end{array}$$

Genes were considered significantly enriched compared to the BS-C soil if the range created by two standard deviations of the mean fold change was entirely > 1 (Su et al., 2015). Genes of different treatments were considered to differ in fold change if their calculated upper and lower ranges did not overlap.

### Prokaryotic Community Analysis Using Illumina MiSeq Sequencing

The prokaryotic community compositions of bulk soil and lettuce rhizosphere samples as well as of the manure used in the experiment were analyzed by Illumina MiSeq sequencing (three replicates each). A fragment of the bacterial 16S rRNA gene was amplified using the primers 341F (5′-CCTAYGGGRBGCASCAG-3′) and 806R (5′-GGACTACNNGGGTATCTAAT-3′) flanking the 460-bp variable V3–V4 region of the target group Prokaryotes including domains of Archaea and Bacteria (Jacquiod et al., 2017). The resulting amplicons were amplified in a second step using the same primers with attached adaptors and barcode tags as previously described (Nunes et al., 2016). The amplification products were purified, and products smaller than 100 bp were removed using the Agencourt AMPure XP beads (Beckman Coulter, Brea, CA, United States) according to the manufacturer’s instructions. The concentrations of the purified amplicons were measured using a Qubit Fluorometer (Life Technologies, Carlsbad, CA, United States). Subsequently, samples were pooled, adjusted to equimolar concentrations, and concentrated using the DNA Clean and Concentrator-5 kit (Zymo Research, Irvine, CA, United States). Finally, the samples were subjected to 2 × 250-bp paired-end high-throughput sequencing on an Illumina^®^ MiSeq^®^ platform (Illumina, San Diego, CA, United States). The obtained sequences were trimmed, clustered, and annotated using a previously described methodology (Nunes et al., 2016). Unassembled raw amplicon data were submitted to the NCBI Sequence Read Archive under the Accession No. PRJNA521325.

The sequences were demultiplexed using the MiSeq Controller software and trimming of the diversity spacers was performed using biopieces ^2^. Mate-pairing and filtering of the sequences was performed using usearch v7.0.1090 (Edgar, 2010). Clustering of OTUs, dereplication, and removal of singletons were performed using uparse (Edgar, 2013). Chimeras were removed using usearch and the ChimeraSlayer package (Haas et al., 2011). Representative sequences for each OTU were defined using Mothur v.1.25.0 with a threshold of 0.8 (Schloss et al., 2009). To build a unifrac phylogenetic tree, Greengenes (DeSantis et al., 2006) with QIIME wrappers for PyNAST (Caporaso et al., 2010a), FastTree (Price et al., 2010), and alignment filtering (Caporaso et al., 2010b) were used. Sequence contingency tables were exported at the species level for bacteria using a 97% similarity threshold. The total number of quality filtered OTUs obtained for each replicate is given in Supplementary Table S2, and the rarefaction curves for each replicate are shown in Supplementary Figure S1.

The prokaryotic community composition between samples was compared by principal component analysis (PCA) using the R package “labdsv” and the pca() function (Roberts, 2016), based on relative abundance of OTUs. For the analysis of differences between manure-treated bulk soil and rhizosphere and the effect of the antibiotic treatments (D0, D50, and D100) as well as their interactions, a permutation test (10,000 permutations) was performed on OTU level with the R package “vegan” (Oksanen et al., 2018) and the function “adonis.” Pairwise distances were calculated using Bray–Curtis dissimilarity. Significant procaryotic responders to the doxycycline treatment at the genus level in bulk soil and lettuce rhizosphere were determined by the R package “edgeR” using the function glmLRT (Robinson et al., 2010; McCarthy et al., 2012). The alpha-diversity indices (Chao’s richness, Pielou’s evenness, and Shannon’s diversity) were calculated as described in Babin et al. (2018). In short, read count data of each sample were 100 times randomly subsampled to the least amount of sequences per sample (*n* = 4,811) and analyzed using the R package “vegan.” Figures showing the relative abundance of OTUs at the phylum and class level were created with the R package “pheatmap” (Klode, 2019).

## Results

### TaqMan-Based Quantification of ARGs and MGEs in Bulk Soil, Rhizosphere, Phyllosphere, and Manure

**FIGURE 1:**
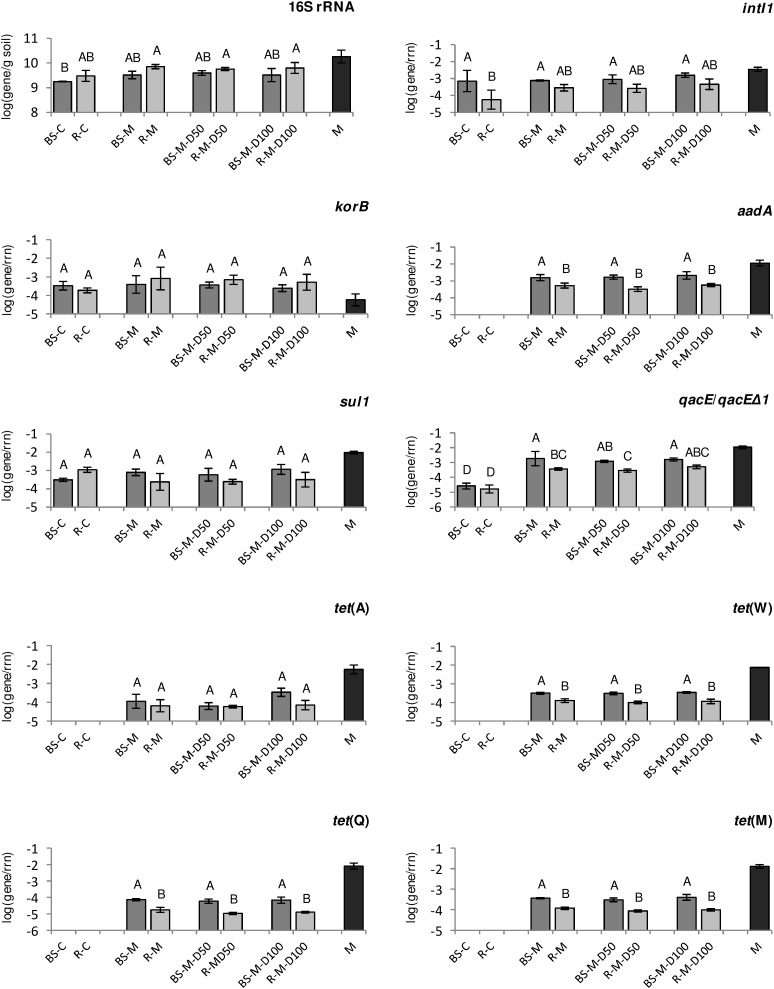
The abundance of the resistance genes, class 1 integron integrase gene, and *korB* specific for IncP-1 plasmids present in lettuce rhizosphere (R, light gray), bulk soil (BS, dark gray), and manure (M, black), obtained by TaqMan-based quantitative PCR (qPCR), relative to 16S rRNA genes (rrn). Error bars indicate the standard deviations of three replicates. Different letters indicate significant differences between soil treatments (*p* < 0.05, Tukey HSD).

In TC-DNA from bulk soil and rhizosphere without manure application (BS-C and R-C), only genes *korB*, *sul1*, *intI1*, and *qacE*/*qacEΔ1* were detected (Figure 1 and Supplementary Table S3). In contrast, *tet*(Q), *tet*(W), *tet*(M), *tet*(A), *qacE*/*qacEΔ1*, *korB*, *aadA*, *sul1*, and integrase gene *intI1* were detected in TC-DNA from all manure-amended soils, except for *intI2*, which was not detected (Figure 1 and Supplementary Table S3). Hence, the relative abundance (relative to 16S rRNA genes) of most of the tested genes was only detectable or considerably increased in bulk soil and rhizosphere after manure application. Accordingly, the tested ARGs as well as integrase genes were detected in the manure sample (Figure 1 and Supplementary Table S3). Lower relative abundances in the manure-treated rhizosphere than in the manure-treated bulk soil samples were observed for the genes *aadA*, *tet*(W), *tet*(M), and *tet*(Q), while no difference was observed between the 16S rRNA gene abundances (Figure 1 and Supplementary Table S3). Although there was a clear manure effect on the relative abundance of ARGs in the bulk soil and rhizosphere, no significant differences were detected between manure-treated soils and soils treated with doxycycline-spiked manure (Figure 1). Accordingly, only the relative abundance of the integrase gene *intI1* in bulk soil showed a weak positive correlation with applied doxycycline concentrations (Pearson correlation coefficient *r* = 0.67, *p* < 0.05, Supplementary Tables S4, S5). However, significant correlations between target genes were observed in both bulk soil and rhizosphere. For instance, the positive correlations between *tet*(Q) and *aadA* (*r* = 0.75 to 0.82) and between *qacE*/*qacEΔ1* and *aadA* (*r* = 0.68 to 0.83) were observed in both compartments, while the positive correlation between *intI1* and *tet*(A) (*r* = 0.77, *p* < 0.01) was only observed in bulk soil but not in the rhizosphere (Supplementary Tables S4, S5). Further differences in correlation coefficients between bulk soil and rhizosphere were observed for *aadA* and *tet*(A) as well as *tet*(W) and *aadA*.

For the lettuce phyllosphere, the absolute abundance of selected target genes, which were sampled from BS-C, BS-M, BS-M-D50, and BS-M-D100, was determined. Most of the tested genes were not detected, with the exception of *tet*(A), which was present in TC-DNA of phyllosphere microbiota from BS-M, BS-M-D50, and BS-M-D100 treatments. The average abundance of *tet*(A) ranged from 6.0 to 9.0 log units (log10 gene copies per gram of leaves).

### High-Throughput Quantitative PCR Quantification of Antibiotic Resistance Genes and Mobile Genetic Elements in Bulk Soil, Rhizosphere, and Manure

**FIGURE 2:**
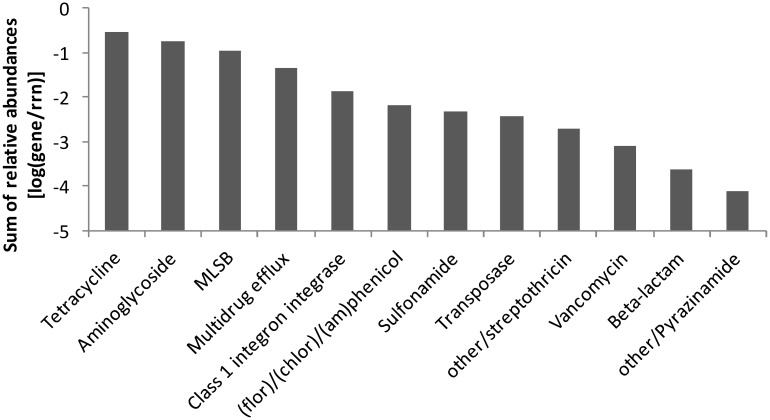
Sum of relative abundances of genes [relative to 16S rRNA genes (rrn)] detected in the manure sample by high-throughput quantitative PCR (HT-qPCR). Genes were sorted by antibiotic and functional classes.

**FIGURE 3:**
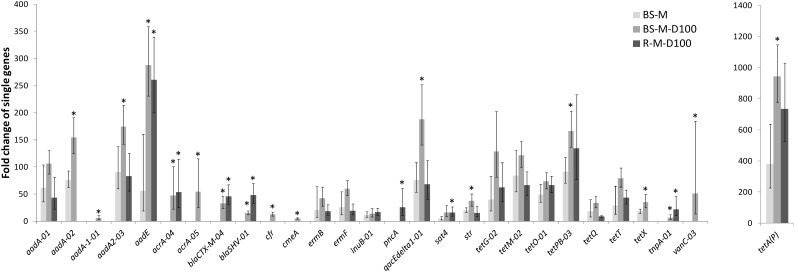
Fold change of genes enriched in BS-M, BS-M-D100, and R-M-D100 compared to BS-C. Shown are the mean values and the corresponding upper and lower ranges of three replicates for bulk soil (BS) and rhizosphere (R), determined by HT-qPCR. Fold changes of genes in BS-M-D100 and R-M-D100 treatments that are higher than the fold changes in the BS-M treatment (not overlapping ranges) are indicated by asterisk.

**FIGURE 4:**
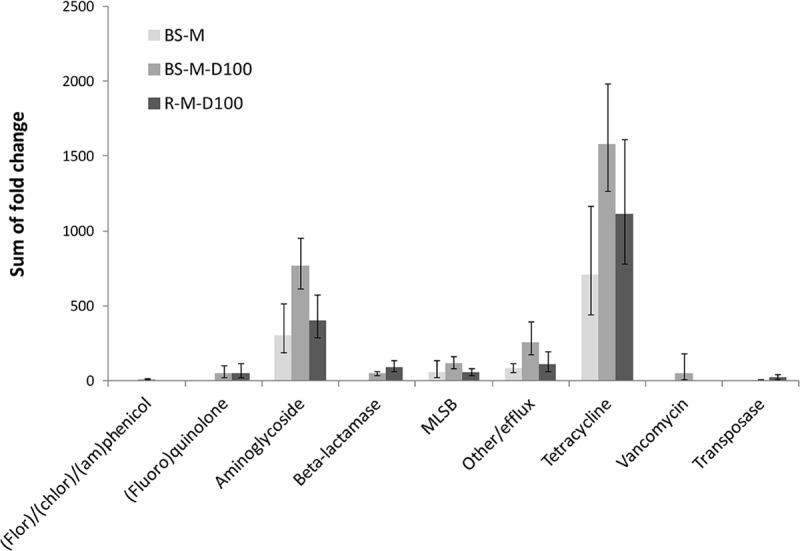
Sums of fold changes of genes enriched in BS-M, BS-M-D100, and R-M-D100 compared to BS-C, obtained by HT-qPCR. Shown are the sums of mean values and the corresponding sums of upper and lower ranges of three replicates for bulk soil (BS) and rhizosphere (R).

In manure, a total of 92 ARGs, MGEs, and class 1 integrons were detected. Tetracycline, aminoglycoside and macrolide–lincosamide–streptogramin B (MLSB) resistance genes as well as multidrug efflux pump-encoding genes showed the highest abundances relative to 16S rRNA genes (Figure 2). The tetracycline resistance genes with the highest proportion of total tetracycline resistance genes tested were *tet*(M), *tet*(T), *tetA*(P), and *tet*(Q) (Supplementary Figure S2). After manure application to soil, a total of 28 ARGs and the transposase gene *tnpA* were significantly enriched in BS-M, BS-M-D100, and/or R-M-D100 compared to BS-C with *tetA*(P), encoding a tetracycline efflux protein, showing the highest enrichment (Figure 3 and Supplementary Table S6). These genes conferred resistance against seven antibiotic classes, namely, aminoglycoside, beta-lactam, (fluoro)quinolone, tetracycline, MLSB, vancomycin, and (flor)/(chlor)amphenicol. Overall, the highest enrichments were observed for the classes of aminoglycoside and tetracycline resistance genes (Figure 4), which were also detected in a high relative abundance in the manure sample (Figure 2).

**FIGURE 5:**
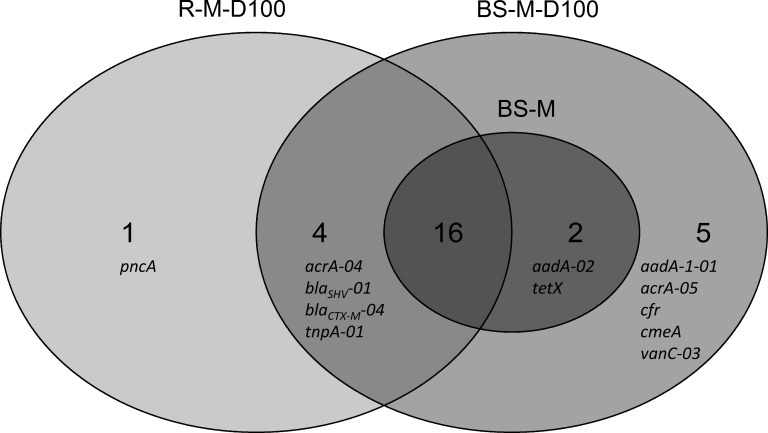
Venn diagram distribution of genes that are enriched on day 42 in the manure and doxycycline-spiked manure treatments (BS-M, BS-M-D100, and R-M-D100) compared to the untreated soil (BS-C), obtained by HT-qPCR.

Furthermore, differences between the manure and doxycycline-spiked manure treatments were observed. In the BS-M, 18 genes were enriched compared to the BS-C. These genes were also detected in the manure sample. This number increased in the doxycycline-spiked manure treatments to 27 and 21 genes in TC-DNA samples from BS-M-D100 and R-M-D100, respectively. A total of 16 enriched genes were simultaneously detected in the BS-M, BS-M-D100, and R-M-D100, while five additional genes were only enriched in the BS-M-D100, one gene was detected only in the R-M-D100, and four genes were solely detected in both the BS-M-D100 and R-M-D100 (Figure 5).

In the BS-M-D100 treatment, the fold change of 17 genes was increased compared to BS-M (Supplementary Table S7). In the rhizosphere, this effect was smaller with only seven and two genes, which were increased and decreased in fold change compared to BS-M, respectively. Furthermore, comparing RH-M-D100 to BS-M-D100, only two genes were higher in fold change, namely, *bla_SHV -01_* and *pncA*, associated with beta-lactam and pyrazinamide resistance, respectively, while the majority of 15 genes had a lower fold change in RH-M-D100 compared to BS-M-D100.

### The Effects of Manure and Doxycycline Application on the Prokaryotic Community Composition in Bulk Soil and Rhizosphere

**FIGURE 6:**
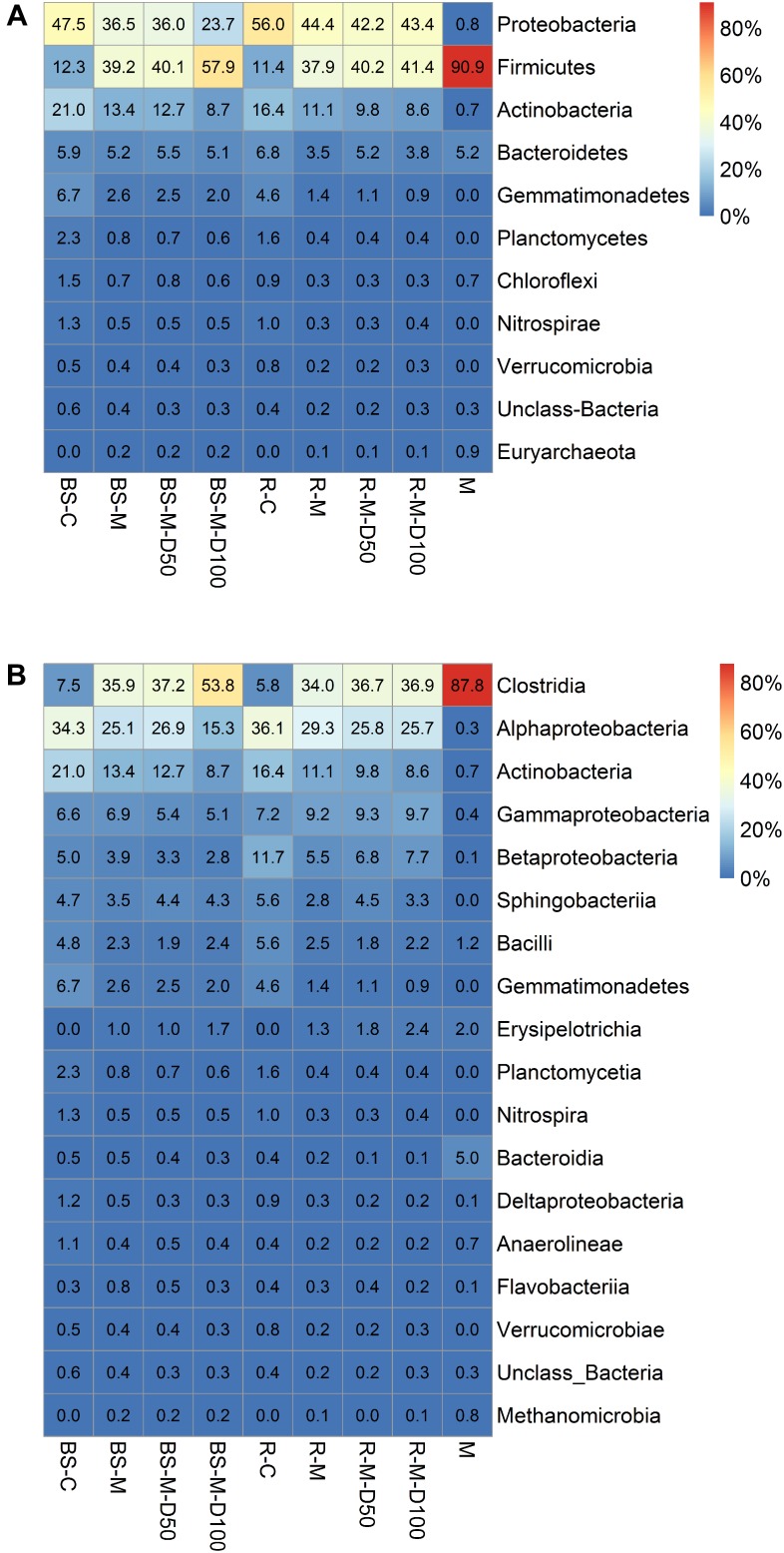
Relative abundance of sequences (only if one value per row > 0.5%) affiliated to prokaryotic phyla **(A)** and classes **(B)** for bulk soil (BS), rhizosphere (R), and manure (M) is shown. Mean values for three replicate bulk soil or rhizosphere samples were calculated. The treatments manure (M), untreated control soil (C), and concentrations of 50 and 100 mg doxycycline kg^-1^ dry soil (D50 and D100) are indicated.

**FIGURE 7:**
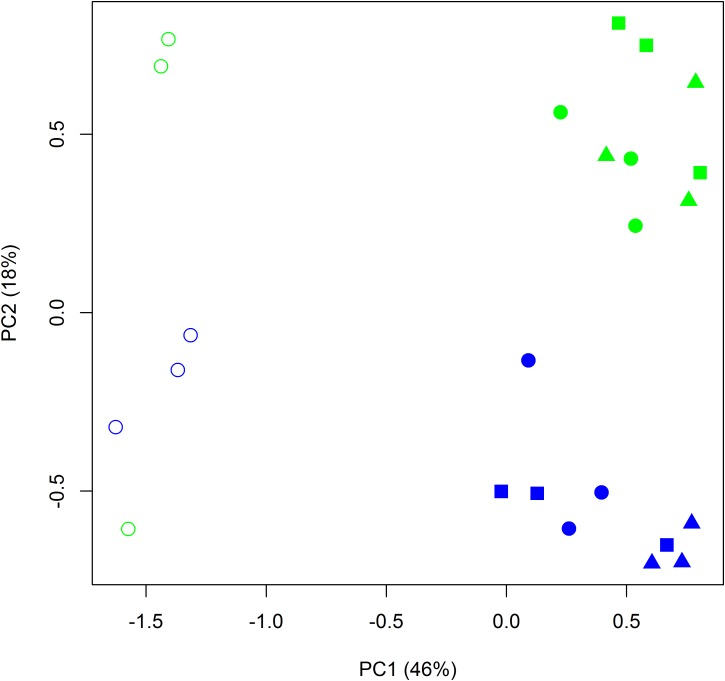
Principal components analysis (PCA) on the log-transformed relative abundance of OTUs obtained from all bulk soil (blue) and rhizosphere (green) samples treated with and without manure (filled and open symbols, respectively), and spiked with doxycycline in low (squares) and high (triangles) concentration or without doxycycline (circles). Principal components (PCs) 1 and 2 explained 46 and 18% of the variance, respectively.

The total number of quality filtered OTUs per replicate is given in Supplementary Table S2. Rarefaction analysis indicated that the obtained sequence numbers were sufficient to assess the prokaryotic diversity in the samples (Supplementary Figure S1). The relative abundances of sequences at the phylum and class levels are shown in Figure 6. In the manure sample, the most abundant class was Clostridia, which was also clearly increased in relative abundance in the BS-M and R-M compared to the BS-C and R-C. Proteobacteria and Actinobacteria were the two most dominant phyla in BS-C and R-C. Alpha-diversity indices showed that the prokaryotic community on day 42 in BS-C and R-C did not differ significantly regarding Shannon diversity index, Pielou’s evenness, or Chao-1 species richness estimate (Supplementary Table S8). Furthermore, manure treatment (BS-M, R-M) did not significantly affect the diversity indices. However, in the BS-M-D100, R-M-D50, and R-M-D100 treatments, the Shannon and Pielou indices were significantly decreased compared to the untreated soils (BS-C and R-C). For the assessment of beta-diversity, the relative abundance of OTUs between samples was further compared by PCA. Here, distinct clusters were observed for BS-C, R-C, BS-M, and R-M (Figure 7).

The prokaryotic community compositions in bulk soil and rhizosphere were significantly different (*p* = 0.046), while the effect of the antibiotic treatment (0, 50, and 100 mg of doxycycline per kilogram of soil applied) was not significant (*p* = 0.245, Table 1). Furthermore, no significant interaction was observed between these two factors (*p* = 0.924).

**Table 1:** Effects of compartment (BS-M and R-M) and doxycycline application (D0, D50, and D100) on soil prokaryotic communities.

Factor	Explained variance %	*p*-Value
Compartment	**13.66**	**0.046**
Doxycycline	14.33	0.242
Compartment × Doxycycline	5.13	0.927
Residual sum	66.88	


*PERMANOVA results are based on Bray–Curtis dissimilarities calculated with OTU counts (10,000 permutations). Bold numbers indicate significant differences (*p* < 0.05).*

For the 50 mg kg^-1^ doxycycline treatment, only three and two significant responders were detected in BS-M-D50 and R-M-D50 compared to BS-M and R-M, respectively (Table 2). In the BS-M-D100 and R-M-D100 treatments, the number of significant responders compared to BS-M and R-M was increased with 12 and 6, respectively (Table 2). Genera that were enriched by doxycycline included *Collimonas* and *Chitinophaga*. The most abundant genera that were decreased in proportion were *Rhizobium* and *Arthrobacter* (Table 2). In R-M, nine genera were significantly enriched, and two genera were significantly decreased compared to BS-M (Supplementary Table S9). Enriched genera with the highest proportions were *Legionella, Sphingobium*, and *Burkholderia*, while *Kineococcus* and *Owenweeksia* were lower in relative abundance in the R-M compared to BS-M. Some of these genera typically include opportunistic human and/or animal pathogens such as *Legionella* and *Yersinia*, which were detected in BS-M at low relative abundance (Table 2 and Supplementary Table S9).

**Table 2:** Bacterial responders at the genus level, calculated with edgeR for bulk soil and rhizosphere spiked with manure and doxycycline 50 (D50) or doxycycline 100 (D100) compared to manured bulk soil or rhizosphere without spiked antibiotics, respectively.

Class	Order	Family	Genus	BS-M		BS-M-D50		BS-M-D100		R-M		R-M-D50		R-M-D100	
				Mean %	SD	Mean %	SD	Mean %	SD	Mean %	SD	Mean %	SD	Mean %	*SD*
Alphaproteobacteria	Rhizobiales	Rhizobiaceae	*Kaistia*	0.07	0.09	0.00^b^	0	0.00^b^	0						
	Rhizobiales	Rhizobiaceae	*Rhizobium*	2.58	3.15			0.55^b^	0.14						
	Rhodobacterales	Rhodobacteraceae	Unclass_ Rhodobacteraceae	0.02	0.01			0.00^b^	0						
	Rickettsiales	Rickettsiaceae	*Orientia*	0	0			0.02^a^	0.01						
	Sphingomonadales	Erythrobacteraceae	*Porphyrobacter*							0.4	0.36			0.06^b^	0
	Rhizobiales	Methylobacteriaceae	*Microvirga*							0.29	0.01			0.08^b^	0.01
Betaproteobacteria	Burkholderiales	Oxalobacteraceae	*Collimonas*	0.13	0.02			0.29^a^	0.09	0.12	0.05	0.40^a^	0.07	0.33^a^	0.1
Gammaproteobacteria	Enterobacteriales	Enterobacteriaceae	*Yersinia*	0.18	0.31	0.00^b^	0								
	Thiotrichales	Thiotrichales_i.s.	*Caedibacter*	0.09	0.12			0.01^b^	0						
	Legionellales	Coxiellaceae	*Diplorickettsia*	0.05	0.05			0.00^b^	0						
Deltaproteobacteria	Bdellovibrionales	Bacteriovoracaceae	*Peredibacter*	0.17	0.07	0.00^b^	0.01	0.01^b^	0.01	0.08	0.05	0.00^b^	0	0.00^b^	0
Actinobacteria	Actinomycetales	Microbacteriaceae	*Salinibacterium*	0.29	0.4			0.05^b^	0.01						
	Actinomycetales	Kineosporiaceae	*Kineococcus*	0.1	0.17			0.00^b^	0						
	Actinomycetales	Micrococcaceae	*Arthrobacter*							2.41	0.32			0.89^b^	0.32
Cytophagia	Cytophagales	Cytophagaceae	*Dyadobacter*	0.17	0.2			0.01^b^	0.01						
Sphingobacteriia	Sphingobacteriales	Sphingobacteriaceae	*Solitalea*	0.01	0.01			0.03^a^	0						
	Sphingobacteriales	Chitinophagaceae	*Chitinophaga*							0.01	0			0.04^a^	0


*The mean relative abundances and standard deviations of three replicates (SD) are indicated. ^*a*^Significantly increased responders. ^*b*^Significantly decreased responders.*

## Discussion

### Manure and Doxycycline Increased the Abundance of Antibiotic Resistance Genes and Mobile Genetic Elements in Bulk Soil

Doxycycline, which is used against a wide range of infections with Gram-negative and Gram-positive bacteria, was frequently detected in manure that was applied as organic fertilizer to agricultural soils (Tylová et al., 2010; Carballo et al., 2016; van den Meersche et al., 2016; Widyasari-Mehta et al., 2016; Wolters et al., 2016; Albero et al., 2018). However, not much is known so far about the effects of doxycycline on the abundance of ARGs and MGEs as well as on the prokaryotic community structure in bulk soil and rhizosphere. In a previous study, we could already show that the application of manure, as well as of doxycycline-spiked manure, to agricultural soils was correlated with an increased relative abundance of ARGs and class 1 integrons. Furthermore, the soil bacterial community composition was affected over a period of 92 days, as revealed by DGGE of 16S rRNA gene fragments amplified from TC-DNA (Blau et al., 2017). Compared to manure, the effect of doxycycline was relatively minor and strongly dependent on the soil texture. However, in the study of Blau et al. (2017), only a limited set of ARGs and MGEs was selected to assess the effects of doxycycline on their relative abundance by TaqMan-based qPCR, together with analysis of the soil bacterial community composition.

In this study, the effect of doxycycline-spiked manure on the relative abundance of ARGs and MGEs, as well as on the prokaryotic community composition, was compared at high resolution between bulk soil and the rhizosphere of lettuce. Therefore, conventional quantification of a selection of 11 genes by TaqMan-based qPCR [genes were selected based on the preceding study of Blau et al. (2017)] was compared to HT-qPCR including 296 genes and combined with Illumina sequencing of 16S rRNA gene amplicons. The application of manure alone drastically increased the abundance of ARGs in bulk soil and rhizosphere as revealed by the qPCR and HT-qPCR assays, confirming the results of previous studies demonstrating an effect of manure application on ARG and MGE abundance in soil (Heuer et al., 2011a; Jechalke et al., 2013b; Hu et al., 2016; Blau et al., 2017; Chen et al., 2017; Zhang et al., 2017; Wolters et al., 2018). The results obtained by TaqMan-based qPCR were in agreement with the HT-qPCR data, both showing a significant increase of *qacE*/*qacEΔ1*, *tet*(Q), *tet*(M), and *aadA* in BS-M compared to BS-C, while additionally *tet*(A) and *tet*(W) were not detected in BS-C but detectable by TaqMan qPCR in BS-M. However, it has to be mentioned that *tet*(W) was not included in the HT-qPCR assay and different primer systems for *tet*(A) were used for the two approaches. The HT-qPCR data showed that mainly tetracycline and aminoglycoside resistance as well as efflux pump-related genes were enriched in BS-M compared to the BS-C. This confirms the results of previous studies demonstrating that the most dominant ARG types in pig manure were tetracycline, aminoglycoside, and MLSB resistance (Zhao et al., 2017; Fang et al., 2018). Likely, one reason for this high abundance of ARGs in pig manure is the presence of in-feed antibiotics and heavy metals that was correlated with ARGs detected in pig gut microbiota (Zhao et al., 2017).

No additional doxycycline effect was observed by TaqMan-based qPCR in soils treated with doxycycline-spiked manure compared to manure-treated soils. Only the relative abundance of the integrase gene *intI1* in bulk soil showed a weak positive correlation with applied doxycycline concentrations. In contrast, a clear effect of the doxycycline treatment was observed by HT-qPCR showing that 17 genes were highly enriched in the BS-M-D100 compared to the BS-M treatment. Importantly, these genes included not only tetracycline resistance genes that were not tested by the TaqMan-based qPCR but also genes conferring resistance to other antibiotic classes as well as the transposon-related gene *tnpA*. These results confirm the results obtained by Blau et al. (2017), showing that doxycycline applied via manure to soil is bioavailable and can select and co-select for ARGs and MGEs in soil bacterial communities. Interestingly, no significant effect of manure or doxycycline-spiked manure application on the abundance of *intI1* genes was observed in this study. We hypothesize that 42 days after application, the effect on *intI1* gene abundance in soil decreased to control levels due to a decrease in manure-derived bacterial populations carrying class 1 integrons over time and due to a decrease in bioavailable concentrations of doxycycline, reducing the selective pressure on soil bacterial communities. This is supported by the study of Blau et al. (2017), observing a lower relative abundance of *intI1* in soil on day 92 compared to day 28 after manure application. Recently, Yan et al. (2018) observed that soil amendment with manure from doxycycline-treated pigs, allowing antibiotic selection already in the animal, increased the number of tetracycline resistance genes *tet*(A), *tet*(M), *tet*(W), and *tet*(X), but not *tet*(G), which was confirmed in this study for *tet*(X) and *tet*(G). Furthermore, the results of the present study demonstrated that the effects of doxycycline entering the soil via manure might be underestimated if only a limited selection of genes is considered in the environmental risk assessment.

### Less Pronounced Effects of Manure and Doxycycline on Antibiotic Resistance Genes and Mobile Genetic Elements in the Rhizosphere of Lettuce

The rhizosphere is regarded as a hotspot of microbial activity and HGT, which might alter the fate and effects of doxycycline in agricultural soils (Rosendahl et al., 2011; Jechalke et al., 2013a,b; Chen et al., 2018). HT-qPCR revealed that the effect of doxycycline-spiked manure on the number of enriched genes and on the fold change of enrichment was less pronounced in the rhizosphere than in bulk soil. This is in agreement with results of previous studies demonstrating smaller effects of antibiotics applied with manure in the rhizosphere (Jechalke et al., 2013a,b; Kopmann et al., 2013; Wang et al., 2015; Kang et al., 2017; Chen et al., 2018). The reduced effect might be related to an accelerated dissipation of doxycycline in the rhizosphere due to plant-enhanced microbial biotransformation processes, as suggested previously for different antibiotics (Rosendahl et al., 2012; Chen et al., 2018). Evidence that the soil microbiome can be involved in doxycycline degradation was given by Yan et al. (2018). However, it cannot be excluded that a higher population density and competition in the rhizosphere buffered the effect of manure and doxycycline application on the abundance of ARGs relative to 16S rRNA gene abundance and decreased the persistence of manure-derived bacteria. This is supported by TaqMan-based qPCR showing lower relative abundances of *aadA*, *tet*(W), *tet*(M), and *tet*(Q) in R-M than in BS-M soil, demonstrating that the smaller effects of doxycycline observed in the rhizosphere are at least partly due to reduced effects of manure on ARG abundance. In contrast, the enrichments of beta-lactam (*bla*) resistance genes, pyrazinamide resistance-associated genes, and transposase genes were similar (*bla_CTX-M_* and *tnpA*) or increased (*bla_SHV_* and *pncA*) in R-M-D100 compared to BS-M-D100. This might suggest that indigenous rhizosphere bacteria carrying *bla* and *pncA* genes might have benefited from manure-derived nutrients and the application of doxycycline. Accordingly, CTX-M enzymes were described to originate in *Kluyvera* spp., which normally inhabit the rhizosphere (Humeniuk et al., 2002; Bevan et al., 2017).

### Distribution of Antibiotic Resistance Genes in the Lettuce Phyllosphere

In TC-DNA obtained from the lettuce phyllosphere, all genes tested by TaqMan-based qPCR, except for *tet*(A), were not detected. This was surprising since previous studies detected ARGs on harvested vegetables grown in soil that was treated with manure or sewage sludge (Marti et al., 2013; Wang et al., 2015; Rahube et al., 2016; Tien et al., 2017). Leaves of field-grown lettuce are exposed to a high number of bacteria originating from dust and soil particles transported by wind and from irrigation water or rainwater, which could already contain bacteria or transfer soil particles to the leaves by splash erosion. Furthermore, bacteria may be transported to crops on-field by different shuttles like insects, livestock, birds, wild animals, and agricultural equipment (Alegbeleye et al., 2018). In the present greenhouse study, it is likely that the phyllosphere did not harbor dense leaf-associated bacterial populations due to controlled conditions, which might explain the low number of ARGs and MGEs detected by qPCR. Therefore, ARGs in the phyllosphere were not analyzed by HT-qPCR. However, *tet*(A) was detected in bacteria from lettuce leaves but only from the manure treatments. This is in agreement with the results of a previous study detecting *tet*(A) in bacteria from the phyllosphere of lettuce grown on manure-amended soil (Wang et al., 2015).

### Manure and Doxycycline Application Affect the Soil Prokaryotic Community Composition

The Illumina sequencing results showed that Actinobacteria and Proteobacteria were the most dominant phyla in BS-C and R-C. Their proportion was highly decreased after manure application. These findings were consistent with several previous studies also reporting a decrease of Actinobacteria after application of organic amendment (Calleja-Cervantes et al., 2015; Liu et al., 2017). At the same time, we observed that manure application increased the proportion of Firmicutes (Clostridia) in bulk soil and lettuce rhizosphere, which was expected regarding the high proportion of Clostridia in manure. This is in line with previous studies demonstrating a high proportion of Clostridia in manure as well as a clear effect on soil bacterial communities (Ding et al., 2014; Jechalke et al., 2014). Leclercq et al. (2016) observed that members of the gut microbiota such as *Clostridium* spp. as well as environmental *Pseudomonas* spp. and *Acinetobacter* spp. were responsible for the persistence of ARGs in manure-amended soils. Furthermore, several previous studies reported that Firmicutes, Proteobacteria, Bacteroidetes, and Actinobacteria represent the dominant phyla in manure and manure-amended soils (Shen et al., 2010; Zhang et al., 2013; Sun et al., 2014; Johnson et al., 2016; Yang et al., 2016; Yan et al., 2018). It was shown that ARGs and in particular tetracycline resistance mechanisms were prevalent in Firmicutes, Proteobacteria, Bacteroidetes, and Actinobacteria (Gibson et al., 2015; Kang et al., 2018), which is in agreement with the observed enrichment of tetracycline resistance genes in manure-treated soils in this study. Interestingly, the tetracycline resistance gene *tetA*(P) was highly enriched in the manure-treated soils and even higher in doxycycline-treated BS-M-D100. This enrichment of the *tetA*(P) gene might have been connected to the enrichment of Clostridia in the manure-amended soil, as this gene was first described in *Clostridium perfringens* (Sloan et al., 1994). Furthermore, these results demonstrate that the *tetA*(P) gene might be a suitable marker for the contamination of soil by ARB, ARGs, and antibiotics originated from manure application.

At the genus level, a clear effect of doxycycline on the prokaryotic soil community was observed that increased with doxycycline concentration. While in BS-M-D50 and R-M-D50 treatments, only three and two responders were identified compared to the BS-M and R-M treatments, respectively, these numbers increased in the BS-M-D100 and R-M-D100 treatments to 12 and 6 responders, respectively. This is in line with the observed reduction in richness and evenness in BS-M-D100, R-M-D50, and R-M-D100 compared to the untreated soils (BS-C and R-C), showing that the evenness of the community was altered due to the increased dominance of some members. A similar doxycycline-concentration-dependent effect on the microbial community composition was observed recently by Yan et al. (2018), applying manure from doxycycline-treated pigs to soil. In agreement with the qPCR data, a smaller number of responders were observed in the rhizosphere, indicating a less pronounced effect of doxycycline on the rhizosphere prokaryotic community. Interestingly, *Collimonas* and *Chitinophaga* were enriched in BS-M-D100, R-M-D50, and R-M-D100. These genera were described to harbor genes encoding degradative enzymes (e.g., chitinase) and secondary metabolites important for interaction with fungi, since these genera have the ability to grow in or on living fungal hyphae (Boer et al., 2004; Song et al., 2015; Shaffer et al., 2017). Therefore, we speculate that an increase in these genera in soil might have been caused by the enrichment of fungal communities, benefiting from the application of doxycycline reducing the number of bacteria competing for nutrients and environmental niches. This would agree with the results of Yan et al. (2018) who observed that the abundance of soil bacteria decreased while the abundance of fungi increased with higher doxycycline concentration. Additionally, genera associated with opportunistic human or animal pathogens, such as *Yersinia* and *Legionella*, were detected in manured soil, which is in line with previous studies (Hong et al., 2013). *Rhizobium* was among the genera that were reduced in abundance in BS-M-D100 compared to BS-M. Some strains belonging to this genus are known to carry *nif* genes involved in the fixation of nitrogen (Shamseldin, 2013), indicating a potential negative effect of doxycycline application on nitrogen fixation of plants.

## Conclusion

The study revealed that the application of manure and manure spiked with doxycycline had a strong impact on the abundance and diversity of ARGs, MGEs, and the prokaryotic community structure, which was more pronounced in the bulk soil than in the rhizosphere of lettuce. This implies that the plant has an important role in modulating the effects and fate of manure-derived antibiotics and ARB, likely via the microbes selected in its rhizosphere by root exudation. Manure application led to a decreased relative abundance of Proteobacteria and Actinobacteria in bulk soil and rhizosphere, while that of Firmicutes increased. Interestingly, the tetracycline resistance gene *tetA*(P) was highly enriched in the manure-treated bulk soil and rhizosphere and highest in BS-M-D100, which might be connected to the increase in Clostridia. These findings demonstrate that the gene *tetA*(P) might be a suitable marker for the contamination of soil by ARB, ARGs, and antibiotics originated from manure application. *Collimonas* and *Chitinophaga* were enriched in the doxycycline treatment, probably associated with an enrichment of fungal communities, which might have benefited from the reduction of bacterial competitors due to the antibiotic treatment applied. The study provided novel insights into the effects of manure containing doxycycline on the bacterial community composition as well as on the abundance of ARGs and MGEs in bulk soil and rhizosphere of lettuce. However, further studies are needed to explore the potential transfer of ARGs and ARB from manure and manured soil to crops and associated risks for human health.

## Author Contributions

KB, KS, and SJe are responsible for research design and concept and wrote the manuscript with contributions of all co-authors. KB and SJe performed the experiments and laboratory work and performed and analyzed the qPCR (TaqMan). SJa and SS performed the Illumina sequencing and processing. SJe, J-QS, and Y-GZ performed the HT-qPCR. SJe analyzed the Illumina sequences and HT-qPCR data.

## Conflict of Interest Statement

The authors declare that the research was conducted in the absence of any commercial or financial relationships that could be construed as a potential conflict of interest. The reviewer JW declared a shared affiliation, with no collaboration, with one of the authors, Y-GZ, to the handling Editor at the time of review.

## Footnotes

1 www.lufa-speyer.de

2 www.biopieces.org
