# Supplementary material for: Manure and Doxycycline Affect the Bacterial Community and Its Resistome in Lettuce Rhizosphere and Bulk Soil
Source: Front Microbiol. 2019 Apr 16;10:725. doi: 10.3389/fmicb.2019.00725 (PMC6477490; doi:10.3389/fmicb.2019.00725)
Supplement: Supplementary file 1 [file Data_Sheet_1.pdf]

Supplementary Material

Supplementary Figures and Tables

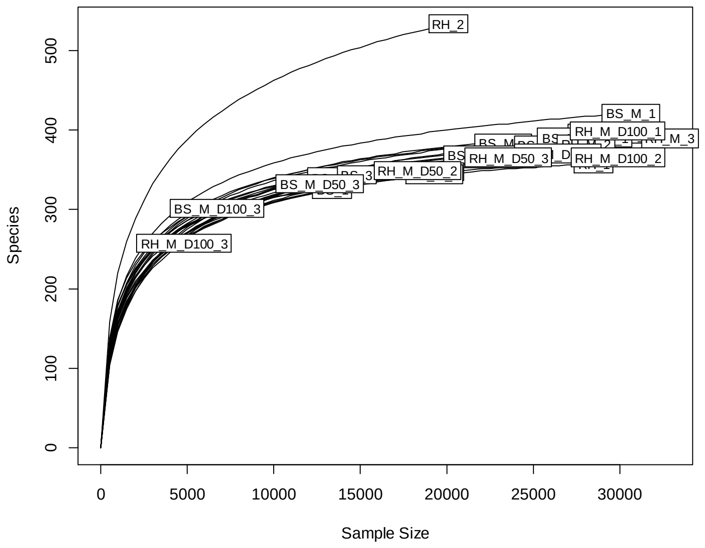

Figure S1: Rarefaction curves of total quality filtered sequences for each replicate. BS: bulk soil, RH: rhizosphere, M: manure, D0/D50/D100: doxycycline 0/50/100 mg kg^{-1} soil. The least amount of sequences per sample was n = 4811.

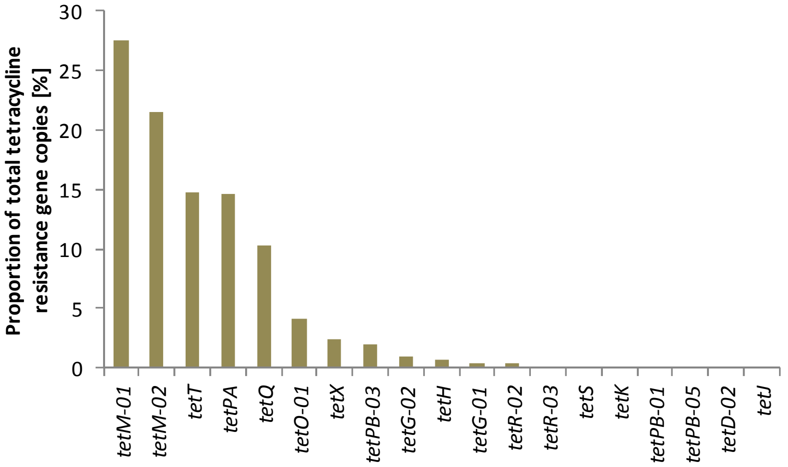

Figure S2: Proportion of tetracycline resistance gene copies in manure relative to sum of all detected tetracycline resistance gene copies. Values were determined by HT-qPCR.

Table S1. qPCR primer sets used in this study.

Gene target	Primer	5'-3' sequence	size (bp)	Reference
qacE/qacEΔ1	qacEall-F	CGCATTTATTTTCTTTCTCTGGTT	69	(Jechalke *et al.*, 2014)
	qacEall-R	CCCGACCAGACTGCATAAGC		
	qacEall-P	TGAAATCCATCCCTGTCGGTGT		
tet(A)	tetA-qfw	CCGCGCTTTGGGTCATT	504	(Guarddon *et al.*, 2011)
	tetA_qfw	TGGTCGCGTCCCAGTGA		
	q-tetA-P	TCGGCGAGGATCG		
sul1	Fq sul-1 653f	CCGTTGGCCTTCCTGTAAAG	965	(Heuer & Smalla, 2007)
	Rq sul-1 719f	TTGCCGATCGCGTGAAGT		
	tp_sul1	CAGCGAGCCTTGCGGCGG		
intI1	intI1-LC1	GCCTTGATGTTACCCGAGAG	196	(Barraud *et al.*, 2010)
	intI1-LC5	GATCGGTCGAATGCGTGT		
	intI1-P	ATTCCTGGCCGTGGTTCTGGGTTTT		
tet(W)	q-tetW-Fw	GCAGAGCGTGGTTCAGTCT	66	(Smith *et al.*, 2004)
	q-tetW-Rv	GACACCGTCTGCTTGATGATAAT		
	q-tetW-p	TTCGGGATAAGCTCTCCGCCGA		
aadA	q-aadA-Fw	TTGATTTGCTGGTTACTGTG'	635	(Walsh *et al.*, 2011)
	q-aadA-Rv	CTTAGTGTGATCTCGCCTTT		
	q-aadA-P	TGGTAGGTCCAGCGGCGGAG		
16s rRNA	Bact1369E	CGGTGAATACGTTCYCGG	460	(Suzuki *et al.*, 2000)
	Prok1492R	GGWTACCTTGTTACGACTT		
	TM1389F	CTTGTACACCGCCCGTC		
kor(B)	KorB-F	TCATCGACAACGACTACAACG'	118	(Jechalke *et al.*, 2013)
	KorB-Fz	TCGTGGATAACGACTACAAACG		
	korB-R	TTCTTCTTGCCCTTCGCCAG		
	korB-Rge	TTYTTCYTGCCCTTGGCCAG		
	korB-Rd	TTCTTGACTCCCTTCGCCAG		
intI2	intI2-LC2	TGCTTTTCCCACCCTTACC'	195	(Barraud *et al.*, 2010)
	intI2-LC3	GACGGCTACCCTCTGTTATCTC'		
	intI2-P	TGGATACTCGCAACCAAGTTATTTTTACGCTG		
tet(Q)	q-tetQ-Fw	AGGTGCTGAACCTTGTTTGATTC'	69	(Smith *et al.*, 2004)
	q-tetQ-Rv	GGCCGGACGGAGGATTT'		
	q-tetQ-P	TCGCATCAGCATCCCGCTC		
tet(M)	tetM-qfw	GGTTTCTCTTGGATACTTAAATCAATCR'	88	(Peak *et al.*, 2007)
	tetM-qrv	CCAACCATAYAATCCTTGTTCRC		
	tetM_tp	ATGCAGTTATGGARGGGATACGCTATGGY		

Table S2. Total number of quality filtered OTUs obtained for each replicate.

Treatment	Replicate 1	Replicate 2	Replicate 3
BS-C	13375	13113	14806
BS-M	30639	23275	25563
BS-M-D50	29559	22680	12663
BS-M-D100	27985	25569	6717
R-C	28503	20087	19867
R-M	19280	28051	31914
R-M-D50	26426	18315	23558
R-M-D100	29878	29914	4811
Manure	17562	na	na

na, not analysed

Table S3. Mean relative abundances of target genes [log(gene/16S rRNA gene)] on day 42 in bulk soil (BS) and lettuce rhizosphere (R) and respective standard deviations (sd) calculated from TaqMan-based qPCR results. The treatments manure (M), untreated control soil (C) and concentrations of 50 and 100 mg doxycycline kg^{-1} dry soil (D50 and D100) are indicated.

Treatment	*intI1*		*aadA*		*qacE/qacE*Δ*1*		*sul1*		*tet*(W)		*tet*(M)		*tet*(Q)		*tet*(A)		*korB*	
	mean	sd	mean	sd	mean	sd	mean	sd	mean	sd	mean	sd	mean	sd	mean	sd	mean	sd
M	-2.46	0.12	-1.95	0.17	-1.97	0.09	-2.02	0.07	-2.13	0.01	-1.89	0.09	-2.09	0.18	-2.26	0.23	-4.24	0.32
BS-C	-3.15	0.63	nd	nd	-4.58	0.20	-3.51	0.40	nd	nd	nd	nd	nd	nd	nd	nd	-3.48	0.23
BS-M	-3.12	0.03	-2.81	0.18	-2.73	0.48	-3.10	0.18	-3.50	0.05	-3.44	0.03	-4.14	0.07	-3.95	0.37	-3.41	0.47
BS-M-D50	-3.05	0.26	-2.78	0.12	-2.91	0.07	-3.23	0.35	-3.51	0.07	-3.52	0.09	-4.23	0.13	-4.20	0.18	-3.44	0.16
BS-M-D100	-2.81	0.13	-2.68	0.22	-2.79	0.10	-2.94	0.27	-3.46	0.04	-3.40	0.14	-4.17	0.19	-3.47	0.22	-3.61	0.18
R-C	-4.25	0.56	nd	nd	-4.78	0.27	-2.96	0.14	nd	nd	nd	nd	nd	nd	nd	nd	-3.73	0.13
R-M	-3.55	0.19	-3.28	0.15	-3.43	0.08	-3.62	0.46	-3.90	0.10	-3.93	0.06	-4.76	0.15	-4.18	0.32	-3.09	0.61
R-M-D50	-3.58	0.24	-3.49	0.14	-3.54	0.11	-3.61	0.13	-4.00	0.07	-4.06	0.05	-4.98	0.07	-4.23	0.07	-3.16	0.25
R-M-D100	-3.34	0.31	-3.25	0.09	-3.29	0.12	-3.50	0.40	-3.94	0.12	-4.01	0.06	-4.90	0.05	-4.15	0.25	-3.29	0.43

Table S4. Pearson correlation coefficient matrix of tested genes and doxycycline concentration (D) in bulk soil.

	D	*intI1*	*qacEΔ1/qacE*	*korB*	*sul1*	*tet(A)*	*tet(Q)*	*tet(M)*	*tet(W)*	*aadA*
D	1.00									
intI1	**0.67***	1.00								
qacEΔ1/qacE	-0.10	0.05	1.00							
korB	-0.27	-0.27	-0.82	1.00						
sul1	0.29	-0.08	0.31	-0.21	1.00					
tet(A)	0.27	**0.77****	0.40	-0.50	-0.18	1.00				
tet(Q)	-0.09	0.41	0.49	-0.20	0.10	0.61	1.00			
tet(M)	0.20	0.58	0.16	0.15	-0.02	0.43	**0.69***	1.00		
tet(W)	0.29	0.51	0.25	-0.11	0.04	0.48	**0.67***	**0.89****	1.00	
aadA	0.34	0.63	**0.68***	-0.83	0.28	**0.84****	**0.75***	0.48	0.53	1.00

* Correlation is significant at the 0.05 level.
** Correlation is significant at the 0.01 level.

Table S5. Pearson correlation coefficient matrix of tested genes and doxycycline concentration (D) in lettuce rhizosphere.

	D	*intI1*	*qacEΔ1/qacE*	*korB*	*sul1*	*tet(A)*	*tet(Q)*	*tet(M)*	*tet(W)*	*aadA*
D	1.00									
intI1	0.15	1.00								
qacEΔ1/qacE	0.36	0.39	1.00							
korB	-0.26	-0.17	-0.42	1.00						
sul1	0.06	-0.56	-0.55	0.66	1.00					
tet(A)	0.28	0.28	0.07	-0.46	-0.54	1.00				
tet(Q)	-0.43	0.36	0.55	-0.23	-0.58	0.17	1.00			
tet(M)	-0.51	-0.23	0.22	0.17	-0.06	0.04	**0.76***	1.00		
tet(W)	-0.17	0.40	**0.77***	-0.20	-0.77	-0.01	0.66	0.34	1.00	
aadA	-0.12	0.38	**0.83****	-0.05	-0.46	-0.07	**0.82****	0.60	**0.82****	1.00

* Correlation is significant at the 0.05 level.
** Correlation is significant at the 0.01 level.

Table S6. HT-qPCR based fold changes (FC) and upper and lower ranges for BS-M, BS-M-D100 and R-M-D100 compared to BS-C.

	BS-M					BS-M-D100					R-M-D100				
gene	deltadeltaCT	ddsd	average FC	upper range	lower range	deltadeltaCT	ddsd	average FC	upper range	lower range	deltadeltaCT	ddsd	average FC	upper range	lower range
aadA-01	-5.94	0.76	61.29	103.72	36.21	-6.73	0.29	106.40	130.04	87.07	-5.42	0.93	42.82	81.52	22.49
aadA-02	-6.25	0.29	76.24	93.13	62.42	-7.27	0.31	154.25	191.03	124.55					
aadA-1-01						-2.45	0.91	5.45	10.25	2.89					
aadA2-03	-6.50	0.60	90.45	137.22	59.62	-7.45	0.29	174.52	213.92	142.38	-6.38	0.58	83.52	125.18	55.73
aadE	-5.81	1.52	55.93	160.56	19.48	-8.17	0.32	287.66	358.60	230.75	-8.03	0.38	260.57	339.25	200.14
acrA-04						-5.56	1.09	47.29	100.64	22.22	-5.74	1.10	53.50	114.66	24.96
acrA-05						-5.77	1.08	54.70	115.47	25.91					
blaCTX-M-04						-5.05	0.49	33.17	46.64	23.58	-5.52	0.55	45.81	67.11	31.27
blaSHV-01						-3.94	0.34	15.38	19.49	12.13	-5.58	0.55	47.72	69.87	32.60
cfr						-3.69	0.42	12.87	17.19	9.63					
cmeA						-2.33	0.37	5.01	6.49	3.88					
ermB	-4.40	1.59	21.18	63.60	7.05	-5.40	0.58	42.16	63.19	28.12	-4.23	0.72	18.72	30.80	11.38
ermF	-4.67	1.11	25.37	54.80	11.75	-5.90	0.32	59.86	74.84	47.87	-4.29	0.71	19.55	32.01	11.94
pncA											-4.68	1.25	25.70	61.15	10.80
qacEdelta1-C	-6.25	0.51	75.94	108.11	53.34	-7.56	0.42	188.15	251.83	140.58	-6.09	0.72	67.94	112.14	41.15
str	-4.31	0.30	19.85	24.49	16.09	-5.24	0.41	37.67	50.01	28.38	-3.93	0.82	15.29	26.95	8.67
tetM-02	-6.40	0.63	84.21	130.36	54.40	-6.92	0.28	121.25	146.86	100.10	-6.05	0.46	66.11	91.13	47.96
tetO-01	-5.62	0.48	49.02	68.15	35.26	-6.19	0.29	73.25	89.43	60.00	-6.04	0.33	66.00	83.09	52.42
tetPA	-8.57	0.74	381.06	638.54	227.41	-9.88	0.28	943.57	1147.31	776.01	-9.52	0.48	735.92	1029.67	525.97
tetPB-03	-6.51	0.37	90.94	117.18	70.58	-7.38	0.28	166.51	202.76	136.74	-7.07	0.80	134.55	233.57	77.50
tetQ	-4.20	1.13	18.33	40.07	8.38	-5.08	0.43	33.76	45.60	25.00	-3.12	0.31	8.70	10.78	7.02
tetT	-4.84	1.17	28.68	64.48	12.76	-6.30	0.32	78.79	98.30	63.16	-5.42	0.42	42.92	57.41	32.09
tetX	-4.18	0.32	18.18	22.71	14.56	-5.13	0.50	34.96	49.32	24.78					
tnpA-01						-2.68	0.89	6.39	11.83	3.45	-4.46	1.05	21.99	45.56	10.62
vanC-03						-5.67	1.86	50.93	184.81	14.04					
tetG-02	-5.33	1.06	40.12	83.44	19.29	-7.01	0.65	128.54	202.31	81.67	-5.96	0.80	62.19	108.22	35.73
sat4	-2.35	0.86	5.10	9.23	2.82	-4.01	0.83	16.10	28.69	9.03	-4.00	0.72	15.95	26.32	9.66
lnuB-01	-3.51	0.63	11.42	17.71	7.37	-3.81	0.73	14.00	23.20	8.44	-4.05	0.55	16.62	24.37	11.33

ddsd: standard deviation (sd) of ΔC_T-values (ddsd = $sd_1^2 + sd_2^2)^{0.5}$)

Empty fields are not significantly different to the BS-C

Table S7. Comparison of enrichments that were calculated based on HT-qPCR results. Higher (+) and lower (-) fold change enrichments are indicated. No significant difference between fold changes of enrichments is indicated by zero.

	compared to BS-M		compared to BS-M-D100
Gene	BS-M-D100	RH-M-D100	RH-M-D100
aadA-01	0	0	-
aadA-02	+	-	-
aadA-1-01	+	0	-
aadA2-03	+	0	-
aadE	+	+	0
acrA-04	+	+	0
acrA-05	+	0	-
blaCTX-M-04	+	+	0
blaSHV-01	+	+	+
cfr	+	0	-
cmeA	+	0	-
ermB	0	0	0
ermF	0	0	-
pncA	0	+	+
qacEdelta1-01	+	0	-
str	+	0	-
tetM-02	0	0	-
tetO-01	0	0	0
tetPA	+	0	0
tetPB-03	+	0	0
tetQ	0	0	-
tetT	0	0	-
tetX	+	-	-
tnpA-01	+	+	0
vanC-03	+	0	-
tetG-02	0	0	0
sat4	0	+	0
lnuB-01	0	0	0
**Total**			
+	17	7	2
-	0	2	15
0	11	19	11

Table S8: Calculation of alpha-diversity indices (Shannon, Pielou, Chao1) based on 100 times randomly subsampled count data (n = 4811). BS: bulk soil, R: rhizosphere, C: control, M: manure, D50/100: doxycycline 50/100 mg kg^{-1} soil. Standard deviations (±) of three replicate samples are shown. Significant differences ($p < 0.05$, Tukey's HSD) between treatments are indicated by different letters.

	Shannon				Pielou				Chao-1			
BS-C	4.49	±	0.08	AB	0.80	±	0.01	A	331	±	8	A
BS-M	4.15	±	0.21	ABC	0.73	±	0.03	AB	356	±	16	A
BS-M-D50	3.95	±	0.50	BC	0.70	±	0.08	AB	337	±	10	A
BS-M-D100	3.51	±	0.19	C	0.63	±	0.04	B	343	±	9	A
R-C	4.63	±	0.13	A	0.81	±	0.01	A	381	±	97	A
R-M	4.01	±	0.01	ABC	0.72	±	0.01	AB	334	±	9	A
R-M-D50	3.92	±	0.27	BC	0.70	±	0.05	AB	331	±	3	A
R-M-D100	3.95	±	0.14	BC	0.71	±	0.03	AB	325	±	27	A

Table S9. Bacterial responder on genus level, calculated with edgeR for manure amended rhizosphere compared to bulk soil without antibiotics spiked. The mean relative abundances and standard deviations of 3 replicates (SD) are indicated.

Class	Order	Family	Genus	BS-M		R-M	
				Mean %	SD	Mean %	SD
Gammaproteobacteria	Legionellales	Legionellaceae	*Legionella*	1.35	0.77	3.85[a]	1.77
Alphaproteobacteria	Sphingomonadales	Sphingomonadaceae	*Sphingobium*	0.78	0.35	4.40[a]	2.82
	Caulobacterales	Caulobacteraceae	*Asticcacaulis*	0.06	0.01	0.36[a]	0.08
	Sphingomonadales	Erythrobacteraceae	*Porphyrobacter*	0.04	0.02	0.40[a]	0.36
Betaproteobacteria	Burkholderiales	Burkholderiaceae	*Burkholderia*	0.74	0.31	2.72[a]	0.23
	Methylophilales	Methylophilaceae	*Methylophilus*	0.13	0.03	0.30[a]	0.05
	Burkholderiales	Oxalobacteraceae	*Herbaspirillum*	0.04	0.02	0.12[a]	0.04
Sphingobacteriia	Sphingobacteriales	Sphingobacteriaceae	*Mucilaginibacter*	0.18	0.13	0.70[a]	0.09
Actinobacteria	Actinomycetales	Kineosporiaceae	*Kineococcus*	0.10	0.17	0.00[b]	0.00
	Actinomycetales	Microbacteriaceae	*Unclass_Microbacteriaceae*	0.06	0.09	1.00[a]	0.91
Flavobacteriia	Flavobacteriales	Cryomorphaceae	*Owenweeksia*	0.04	0.01	0.00[b]	0.00

a: significantly increased responders
b: significantly decreased responders

References

Barraud O, Baclet MC, Denis F & Ploy MC (2010) Quantitative multiplex real-time PCR for detecting class 1, 2 and 3 integrons. *J Antimicrob Chemother* **65:** 1642–1645.

Guarddon M, Miranda JM, Rodriguez JA, Vazquez BI, Cepeda A & Franco CM (2011) Real-time polymerase chain reaction for the quantitative detection of tetA and tetB bacterial tetracycline resistance genes in food. *Int J Food Microbiol* **146:** 284–289.

Heuer H & Smalla K (2007) Manure and sulfadiazine synergistically increased bacterial antibiotic resistance in soil over at least two months. *Environ Microbiol* **9:** 657–666.

Jechalke S, Dealtry S, Smalla K & Heuer H (2013) Quantification of IncP-1 plasmid prevalence in environmental samples. *Appl Environ Microbiol* **79:** 1410–1413.

Jechalke S, Schreiter S, Wolters B, Dealtry S, Heuer H & Smalla K (2014) Widespread dissemination of class 1 integron components in soils and related ecosystems as revealed by cultivation-independent analysis. *Front Microbiol* **4:** 420.

Peak N, Knapp CW, Yang RK, Hanfelt MM, Smith MS, Aga DS & Graham DW (2007) Abundance of six tetracycline resistance genes in wastewater lagoons at cattle feedlots with different antibiotic use strategies. *Environ Microbiol* **9:** 143–151.

Smith MS, Yang RK, Knapp CW, Niu Y, Peak N, Hanfelt MM, Galland JC & Graham DW (2004) Quantification of tetracycline resistance genes in feedlot lagoons by real-time PCR. *Appl Environ Microbiol* **70:** 7372–7377.

Suzuki MT, Taylor LT & DeLong EF (2000) Quantitative analysis of small-subunit rRNA genes in mixed microbial populations via 5'-nuclease assays. *Appl Environ Microbiol* **66:** 4605–4614.

Walsh F, Ingenfeld A, Zampicolli M, Hilber-Bodmer M, Frey JE & Duffy B (2011) Real-time PCR methods for quantitative monitoring of streptomycin and tetracycline resistance genes in agricultural ecosystems. *J Microbiol Methods* **86:** 150–155.
